# Aquaporin-3 positively regulates matrix metalloproteinases via PI3K/AKT signal pathway in human gastric carcinoma SGC7901 cells

**DOI:** 10.1186/1756-9966-30-86

**Published:** 2011-09-25

**Authors:** Hao Xu, Yong Xu, Wenjie Zhang, Lizong Shen, Li Yang, Zekuan Xu

**Affiliations:** 1 Department of General Surgery, First Affiliated Hospital of Nanjing Medical University, 300 Guangzhou Road, Nanjing, Jiangsu Province, 210029, China

**Keywords:** Aquaporin-3, Matrix metalloproteinase, PI3K/AKT pathway

## Abstract

**Background:**

matrix metalloproteinases (MMPs) are produced by tumor cells, so they may be associated with tumor progression including invasion, migration, angiogenesis and metastasis. Aquaporin-3 (AQP3) also plays a critical role in gastric cancer cell migration and proliferation.

**Methods:**

In this study, AQP3 was silenced or over-expressed in SGC7901 cells.

**Results:**

We found a significant decrease in MT1-MMP, MMP-2, and MMP-9 expression after AQP3 knockdown, and a significant increase in MT1-MMP, MMP-2, and MMP-9 expression after AQP3 over-expression in SGC7901 cells. We also found that AQP3 silence led to a significant decrease of phosphorylation of ser^473 ^in AKT in SGC7901 cells.

**Conclusion:**

Our findings showed that AQP3 might positively regulate MMPs proteins expression through PI3K/AKT signal pathway in human gastric carcinoma SGC7901 cells.

## Background

Gastric cancer (GC) remains a major cause of mortality and morbidity worldwide [1]. The rapid invasion and metastasis of tumor cells are responsible for poor prognosis [2]. The high expression of MMPs in GC tissues has been determined in several studies [3,4]. It has shown that MMPs expression correlates with clinical pathological features of GC, such as tumor stage, depth of tumor invasion and the presence of lymph node and distant metastases [5].

Aquaporins (AQPs) are a family of small (30 kDa/monomer) hydrophobic, integral membrane proteins, which belong to a special superfamily of membrane integral proteins called MIPs (major intrinsic proteins) [6,7]. In our previous work, we showed a differential expression of AQPs between human gastric carcinomas and corresponding normal tissue, and the association of AQP3 expression with the lymph node metastasis and lymphovascular invasion of human gastric carcinoma [8].

The PI3K signal pathway plays an integral role in many normal cellular processes, including survival, proliferation, differentiation, metabolism and motility, in a variety of cell types. Although a number of studies have convincingly demonstrated that the PI3K/AKT pathway regulated MT1-MMP activity,[9] but the molecular mechanisms are still unclear. Here, we reported that AQP3 positively regulated MMPs proteins expression through PI3K/AKT signal pathway in human gastric carcinoma cells.

## Materials and Methods

### Cell culture

Human gastric cancer cell line (SGC7901) were kindly provided by Shanghai Institute of Cell Biology, Chinese Academy of Sciences (Shanghai, China) and were grown in DMEM supplemented with 10% fetal bovine serum (FBS), 100 μg/ml streptomycin and 100 units/ml penicillin at 37 °C in a humidified incubator in an atmosphere of 5% CO_2_.

### Antibodies and reagents

Rabbit anti-AQP3 antibody was obtained from Santa Cruz Biotechnology (Santa Cruz, CA). Antibodies against total AKT, Ser^473 ^phosphorylated AKT, and β-actin were supplied by Cell Signaling Technology(Beverly, MA, USA). Lentiviral vectors encoding AQP3 and the shRNA (more than four sequences) for AQP3 were designed and chemically synthesized by Genephama Biotech(Shanghai, China). LY294002, MT1-MMP, MMP-2, and MMP-9 antibodies were purchased from Abcam (Hong Kong, China).

### Lentiviral transfection

ShRNA of human AQP3 lentivirus gene transfer vector encoding green fluorescent protein(GFP) and puromycin sequence was constructed by Genephama Biotech(Shanghai, China). The lentiviral-scrambled-shRNA served as negative control. For shRNA of human AQP3, the oligonucleotide sequences were GGCTGTATTATGATGCAATCT. The aqp3shRNA was packaged with lentivirus following the manufacturer's protocols. When SGC7901 cells grew to 60-70% confluence, the cells were infected with lentiviral-scrambled-shRNA or lentiviral vector encoding AQP3 at a multiplicity of infection (MOI) of 20. Stable cell lines were selected with 2 μg/ml puromycin (Sigma-Aldrich) for one week. After that, cells were analyzed using quantitative RT-PCR and Western blot for AQP3 expression.

### Reverse transcription and real-time PCR

Total RNA was extracted from the cells using the Trizol reagent (Invitrogen, Carlsbad, Calif) and exactly following the manufacturer's manual under RNase-free condition. After complementary DNA was synthesized with a two-step reverse transcription reaction kit(TAKARA, Dalian, China), quantitative PCR was performed on an Applied Biosystems 7500 Real-time PCR System using SYBR Premix Ex Taq Kit (TAKARA, Dalian, China) in Axygen 96-well reaction plates following the manufacturer's protocols. β-actin was used as a reference to obtain the relative fold change for target samples using the comparative Ct method. The primers used are as follows: β-actin forward, TCACCCACACTGTGCCCATCTACGA; β-actin reverse, CAGCGGAACCGCTCATTGCCAATGG, AQP3 forward, CACAGCCGGCATCT- TTGCTA, reverse, TGGCCAGCACACACACGATA, All cell preparations and real-time PCRs were performed in triplicate.

### Western blot analysis

For Western blot, cells were reseeded in 6-well plates at a density of 0.2 × 10^6 ^cells/ml with fresh complete culture medium. Cells with or without treatment were washed with cold PBS and harvested by scraping into 150 μl of RIPA buffer(containing 50 mM Tris-HCl, pH 7.4, 150 mM NaCl, 1% NP-40, 1 mM EDTA 0.25% sodium deoxycholate) with 1mM NaF, 10 μM Na3VO4, 1 mM PMSF, and a protease inhibitor concoction(10 μg/ml leupeptin, 10 μg/ml aprotinin, and 1 μM pepstatin). Cell lysates were incubated at 4°C for 30 min. After centrifugation at 12,000 rpm for 20 min at 4°C, protein concentrations were determined by bicinchoninic acid(BCA) protein assay. Forty micrograms of proteins(for AQP3, MT1-MMP, MMP-2, MMP-9, phospho-AKT or AKT) were denatured in 5× SDS-PAGE sample buffer for 5 min at 100°C. The proteins were separated by 12% SDS-PAGE and transferred onto PVDF membrane(Millipore, Bedford, MA) for 90 min at 4°C. Nonspecific binding was blocked with 5% dry skimmed milk in TBST (20 Mm Tris-HCl, 137 mM NaCl, 0.1% Tween 20, pH 7.4) for 2 h at room temperature. After blocking, membranes were incubated with specific antibodies against AQP3(1:500), MT1-MMP(1:1,000), MMP-2(1:1,000), MMP-9(1:1,000), phospho-AKT(1:1,000), or AKT(1:1,000) in dilution buffer (2% BSA in TBS) overnight at 4°C. The blots were incubated with HRP-conjugated anti-mouse or anti-rabbit IgG (1:2,000) at room temperature for 2 h. Antibody binding was detected using an enhanced chemiluminescence(ECL) detection system following manufacturer's instructions and visualized by autoradiography with Hyperfilm. Semiquantitatively analyzed of the blots were acquired using the software Quantity One(BioRad, USA). The density for AQP3, MMPs, or phospho-AKT protein in their parental sample was normalized to 1.0, and the values for other treatments were calculated against this value.

### Statistical analysis

All data were expressed as mean ± SD. Statistical analyses were performed using Student's t test or analysis of variance (ANOVA). The values of P < 0.05 are considered significant.

## Results

### AQP3 expression in SGC7901 cells after lentiviral transfection

**Figure 1:**
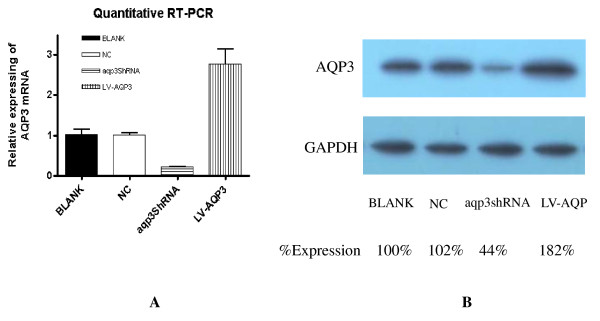
**The expression level of AQP3 in SGC7901 in real-time PCR and Western blot studies**. AQP3 mRNA and protein were expressed in SGC7901 cells. After RNAi, both AQP3 mRNA and protein expression decreased significantly. After transfection of lentivector encoding AQP3, both AQP3 mRNA and protein expression levels were increased obviously. The expression levels of different cells were further normalized to that of BLANK group, making the relative expression level of BLANK group as 100%.

We examined the expression of AQP3 at its mRNA and protein levels by performing quantitative RT-PCR and Western blot analysis in SGC7901 cells. After transfection of aqp3shRNA, stable cell lines were harvested for quantitative RT-PCR and Western blot analysis. After transfection of lentiviral vector encoding AQP3, cells were collected for quantitative RT-PCR and Western blot analysis too. AQP3 mRNA and protein were expressed in SGC7901 cells. After RNAi, both AQP3 mRNA and protein expression decreased significantly. After transfection of lentiviral vector encoding AQP3, both AQP3 mRNA and protein expression increased obviously. (Figure 1)

### AQP3 silence down-regulated MMPs expression in SGC7901 cells

**Figure 2:**
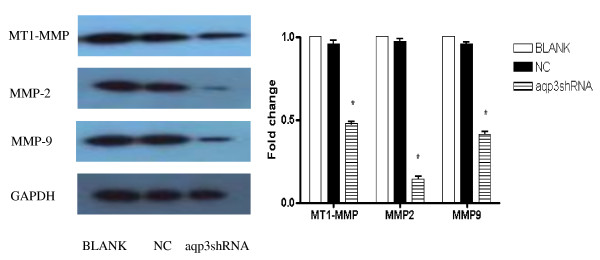
**AQP3 regulated MMPs expression in SGC7901 cells**. AQP3 silence down-regulated MMPs expression in SGC7901 cells. AQP3 regulated MMPs expression in SGC7901 cells. AQP3 silence down-regulated MMPs expression in SGC7901 cells. A significant decrease in MT1-MMP, MMP-2, MMP-9 expression was observed in AQP3 knockdown group compared with control group.* p < 0.05 BLANK control SGC7901 cells NC cells treated with scrambled shRNA aqp3shRNA cells treated with aqp3shRNA

The levels of MT1-MMP, MMP-2, and MMP-9 protein expression were detected by Western blot analysis. A significant decrease in MT1-MMP, MMP-2, and MMP-9 expression was observed in AQP3 knockdown group compared with control group. (Figure 2)

### AQP3 over-expression up-regulated MMPs expression in SGC7901 cells

**Figure 3:**
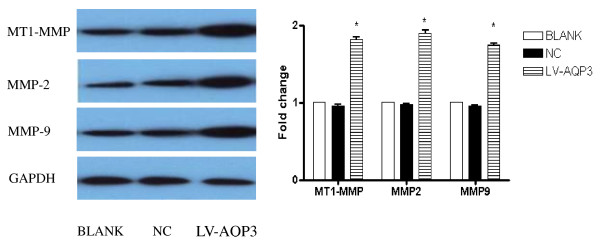
**AQP3 regulated MMPs expression in SGC7901 cells**. AQP3 over-expression up-regulated MMPs expression in SGC7901 cells. A significant increase in MT1-MMP, MMP-2, MMP-9 expression was observed in AQP3 over-expression group compared with control group.* p < 0.05 BLANK control SGC7901 cells NC cells treated with scrambled shRNA LV-AQP3 cells treated with lentiviral vector encoding AQP3

The levels of MT1-MMP, MMP-2, and MMP-9 protein expression were detected by Western blot analysis. A significant increase in MT1-MMP, MMP-2, and MMP-9 expression was observed in AQP3 over-expression group compared with control group. (Figure 3)

### AQP3 silence blocked PI3K/AKT pathway in SGC7901 cells

**Figure 4:**
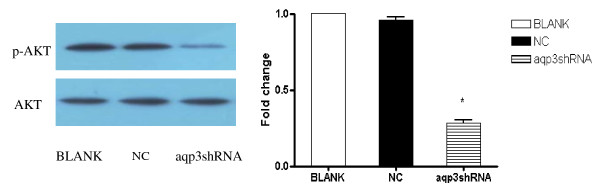
**AQP3 regulated PI3K/AKT pathway in SGC7901 cells**. AQP3 silence blocked PI3K/AKT pathway in SGC7901 cells. AQP3 silence led to a significant decrease in phosphorylation of ser^473 ^in AKT. * p<0.05 BLANK control SGC7901 cells NC cells treated with scrambled shRNA aqp3shRNA cells treated with aqp3shRNA

To determine whether the PI3K/AKT pathway was involved in the AQP3 silence down-regulated MMPs expression SGC7901 cells, we first compared levels of phosphorylated and total AKT in SGC7901 cells treated with AQP3 interference by using Western blot. AQP3 silence led to a significant decrease in phosphorylation of ser^473 ^in AKT. (Figure 4)

### AQP3 up-regulation activated PI3K/AKT pathway in SGC7901 cells

**Figure 5:**
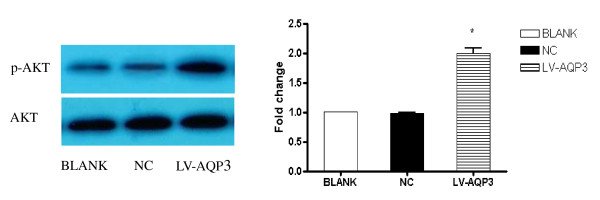
**AQP3 regulated PI3K/AKT pathway in SGC7901 cells**. AQP3 over-expression activated PI3K/AKT pathway in SGC7901 cells. AQP3 over-expression led to a significant increase in phosphorylation of ser^473 ^in AKT. * p < 0.05 BLANK control SGC7901 cells NC cells treated with scrambled shRNA LV-AQP3 cells treated with lentiviral vector encoding AQP3

We compared levels of phosphorylated and total AKT in SGC7901 cells with AQP3 over-expression by using Western blot. AQP3 over-expression led to a significant increase in phosphorylation of ser^473 ^in AKT. (Figure 5)

### LY294002 down-regulated MMPs expression in SGC7901 cells

**Figure 6:**
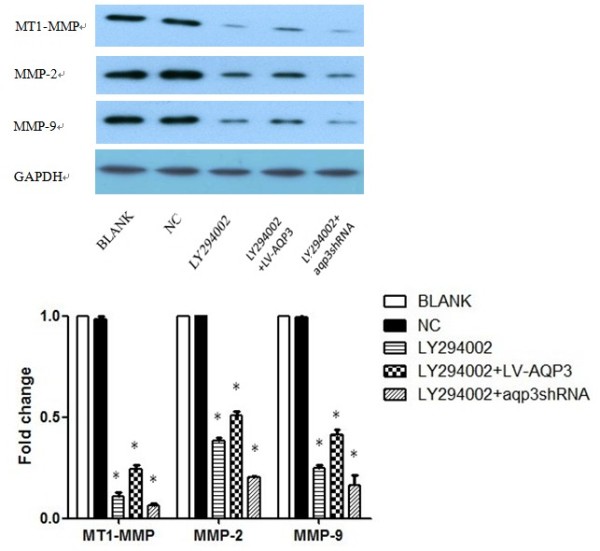
**LY294002 down-regulated MMPs expression and blocked the effect of LV-AQP3 and aqp3shRNA in SGC7901 cells**. SGC7901 cells were exposed to LY294002 for 48h and then were harvested to perform Western blot analysis. We found a significant decrease in MT1-MMP, MMP-2, and MMP-9 expression. However, with the addition of LY294002, the expression of MMPs could not be obviously reversed in LV-AQP3 or aqp3shRNA groups. * p < 0.05 BLANK control SGC7901 cells NC cells treated with scrambled shRNA LY294002 cells treated with LY294002 LY294002+LV-AQP3 cells treated with LY294002 and LV-AQP3 LY294002+aqp3shRNA cells treated with LY294002 and aqp3shRNA

SGC7901 cells were exposed to 20 μM LY294002 for 48 h (fresh media containing LY294002 was added every 24 h), and then were harvested to perform Western blot. We found a significant decrease in MT1-MMP, MMP-2, and MMP-9 expression. However, with the addition of LY294002, the expression of MMPs could not be obviously reversed in LV-AQP3 or aqp3shRNA groups. (Figure 6)

## Discussion

Recent studies showed that the involvement of AQPs in angiogenesis and tumor cell migration and proliferation had potentially important clinical implication [10,11]. We reported for the first time that AQP4 protein and mRNA expression levels in gastric cancer tissue were significantly lower than those in normal gastric tissue [12]. Then, we demonstrated that AQP3 played a critical role in gastric cancer cell migration and proliferation in previous study [13]. In this study, we found that AQP3 silence could down-regulate MMPs expression and AQP3 over-expression could up-regulate MMPs expression in SGC7901 cells.

Many tumors exhibit elevated levels of MMPs, which may play an important role in cellular invasion and metastasis [14]. Among the human MMPs reported to date, MT1-MMP, MMP-2 and MMP-9 are the major enzymes involved in degrading types I and IV collagen and the extracellular matrix(ECM) [15]. Tumor-secreted MMPs destroy the ECM components in tissues surrounding a tumor, enabling tumor cells to pass through the basement membrane of blood vessels and facilitating their spread to distant organs, resulting in organ failure and patient mortality. MT1-MMP, MMP-2 and MMP-9, which are abundantly expressed in various malignant tumors, contribute to cancer invasion and metastasis [15].

In our study, AQP3 over-expression could up-regulated MMPs expression in SGC7901 cells. Hwang et al. and Kajanne et al. indicated that MMPs could be stimulated by an inflammatory cytokine, epidermal growth factor (EGF), through the activation of different intracellular signal pathways [16,17]. This was consistent with our results. We supposed that AQP3 might be involved in MMPs stimulatory pathway in SGC7901 cells.

PI3K/AKT signal pathway was found abnormally activated and closely associated with tumorigenesis and tumor progression [18]. AKT is a key regulator of cell survival and apoptosis, increased AKT phosphorylation has been reported in a variety of cancers [19]. Our results showed that AKT was phosphorylated excessively and AQP3 silence led to a significant decrease in phosphorylation of ser473 in AKT in SGC7901 cells. LY294002 is a specific inhibitor of PI3K, and is generally used in research on PI3K/AKT signal pathway. After treatment with LY294002, the p-AKT expression levels in SGC7901 cells decreased obviously, suggesting its high performance in blocking PI3K/AKT signal pathway by suppressing AKT phosphorylation catalyzed by PI3K. Meanwhile, LY294002 could decrease MT1-MMP, MMP-2, and MMP-9 expression in SGC7901 cells. However, with the addition of LY294002, the expression of MMPs could not be obviously reversed in LV-AQP3 or aqp3shRNA groups. And this result is a further evidence of the involvement of PI3K/AKT pathway in AQP3 regulating MMPs.

In conclusion, our findings emphasize that AQP3 might up-regulate MMPs proteins expression via the PI3K/AKT signal pathway in human gastric carcinoma SGC7901 cells.

## Competing interests

The authors declare that they have no competing interests.

## Authors' contributions

HX and YX carried out the molecular genetic studies and drafted the manuscript. WJZ carried out the transfection. LZS and LY performed the statistical analysis. HX participated in the design of the study. ZKX conceived of the study, and participated in its design and coordination. All authors read and approved the final manuscript.
